# The Effects of Climate on Tubercular Disease; The Influence of Pregnancy on the Development of Tubercles

**Published:** 1857-11

**Authors:** 


### BOOK NOTICES.

### FISK FUND PRIZE ESSAYS.

*The Effects of Climate on Tubercular Disease. By Edwin Lee, M.R.C.S., London. Being the Dissertation to which the Fisk Fund Prize was awarded, June 6th,* 1855.

*The Influence of Pregnancy on the Development of Tubercles. By Edward Warren, M.D., of Edenton, N. C. Being the Dissertation to which the Fisk Fund Prize was awarded, June 4th,* 1856.

"Dr. Caleb Fisk, who was President of the Rhode Island Medical Society in 1823 and 1824, at his death bequeathed to that Society a fund of two thousand dollars, directing the annual income to be expended in premiums for essays on subjects selected for competition. The first premium of forty dollars was awarded June 27th, 1836, since which time a large number of valuable dissertations have been laid before the profession through the instrumentality of Dr. Fisk's well directed munificence. By the judicious management of the trustees the fund has gradually increased, and they are now able to offer two annual prizes of one hundred dollars each." (Preface.)

Innocent entirely so far as we can see of originality, Mr. Lee's essay is a fair summary of our knowledge of the effects of climate upon tuberculous patients. Being very short, and tolerably well written, it will bear reading. It is orthodox pretty thoroughly, in everything except the assertion in relation to the ignorance of American physicians on auscultation. The absurd statement that the "exploration of the state of the organs contained in the thoraic cavity, by means of auscultation and percussion, forms no part of the education of medical students" in this country, which he makes on page 19, ought to have shut the eyes of any American committee against its further perusal. Such a statement made to an American awarding committee exhibits a degree of stupidity that could exist nowhere but in England, and is equaled only by the submissive assent of the Fisk fund prize essay committee by tolerating it in a prize paper.

Dr. Warren's paper shows much ingenuity and considerable research. He takes the ground that in gestation as a consequence and requisite, every function necessary to a higher state of general health is performed in as perfect a manner as possible, and thus organic lesion of almost every kind is prevented, and if in existence, either cured or rendered stationary. He thinks that consumption consists essentially in a low and depraved condition of the nutritive functions, and that the condition of the system in gestation being opposite in its character, more surely counteracts and arrests tuberculosis than any other serious diathesis or disease. It may be remarked, however, in this connection, that this is merely an arrest and amelioration, when it does occur, and not (at least, it is not often) curative in the strict sense of the word. Too many cases have fallen under the notice of the writer in which consumptive patients were apparently very much better during gestation, and after confinement rapidly sank under the ravages of their disease and died, not to warn him against a favorable prognosis in most cases. The essay will well repay perusal. The authors therein cited, if the theory of Dr. Warren in regard to the nature of phthisis is correct, all seem to favor the general tenor of the essay, and the number and respectability of them are highly creditable as company in any opinion connected with the physiology and pathology of pregnancy.

